# Comorbidities, Routine Biomarkers and Early Septic Shock Occurrence in Patients with Sepsis: A Retrospective Longitudinal Study

**DOI:** 10.3390/diagnostics16091334

**Published:** 2026-04-29

**Authors:** Jasna Petrović

**Affiliations:** 1 Centar Valjevo, University Singidunum, Danijelova 23, 11000 Belgrade, Serbia; pjassna@gmail.com; 2 Intensive Care Unit, Department of Anesthesia, General Hospital “Valjevo”, Obrena Nikolića 5, 14000 Valjevo, Serbia

**Keywords:** sepsis, septic shock, comorbidity, biomarkers, LASSO logistic regression, linear mixed-effects models, longitudinal analysis

## Abstract

**Background/Objectives**: Early recognition of septic shock among patients with sepsis remains clinically important, particularly in settings where risk stratification relies on routinely available indicators. This study aimed to identify patient-related factors associated with septic shock, with particular emphasis on comorbidities, and to examine early changes in selected inflammatory markers during the first 72 h after diagnosis. **Methods**: This study included 106 intensive care patients diagnosed with sepsis or septic shock according to Sepsis-3 criteria. Baseline demographic, clinical, and laboratory characteristics were compared between groups using univariate tests. Candidate predictors were then evaluated using a LASSO-assisted logistic regression model. Longitudinal changes in inflammatory markers were analyzed using linear mixed-effects models. **Results**: Among baseline inflammatory markers, only procalcitonin was significantly higher in patients with septic shock than in those with sepsis. In the final logistic regression model, higher procalcitonin levels were associated with greater odds of septic shock, whereas gastrointestinal comorbidity was associated with lower odds. Patients older than 75 years also had significantly higher odds of septic shock. The model showed satisfactory discrimination (AUC = 0.795). During the first 72 h, only neutrophil percentage demonstrated a significantly different temporal pattern between groups. **Conclusions**: Early recognition of septic shock may benefit from combining routine biomarkers with patient-related clinical characteristics rather than relying on isolated laboratory measurements alone. Procalcitonin, older age, gastrointestinal comorbidity, and early neutrophil dynamics emerged as the most informative signals, although these findings require validation in larger multicenter cohorts.

## 1. Introduction

Sepsis remains one of the major global health challenges and a leading cause of morbidity and mortality among critically ill patients worldwide. According to recent global estimates, sepsis affects approximately 49 million individuals annually and is associated with around 11 million deaths, accounting for nearly 20% of all deaths worldwide [1]. Under the Sepsis-3 definition, sepsis is described as a life-threatening organ dysfunction caused by a dysregulated host response to infection [2]. This concept emphasizes that sepsis is not merely the presence of infection, but a complex and potentially fatal clinical syndrome resulting from an abnormal host reaction that leads to acute organ impairment.

Historically, the definition of sepsis has undergone substantial evolution, from the first consensus conference held in 1991 (Sepsis-1) [3], through the 2001 revision (Sepsis-2) [4], to the Sepsis-3 recommendations published in 2016 [2]. In current clinical practice, the diagnosis of sepsis is based on the assessment of organ dysfunction using the Sequential Organ Failure Assessment (SOFA) score [2]. An increase in the SOFA score of 2 points or more is considered indicative of clinically relevant organ dysfunction and is associated with an increased risk of in-hospital mortality. Septic shock is recognized as a more severe subset of sepsis, characterized by profound circulatory, cellular, and metabolic abnormalities and a substantially higher risk of death [5]. Clinically, it is defined by the need for vasopressor therapy to maintain a mean arterial pressure of at least 65 mmHg together with a serum lactate level above 2 mmol/L despite adequate fluid resuscitation [5]. The distinction between sepsis and septic shock is of major clinical importance, as it reflects differences in severity, prognosis, and treatment urgency.

Because sepsis is biologically heterogeneous, biomarkers are used primarily as supportive rather than stand-alone tools, and no single biomarker has shown sufficient diagnostic and prognostic accuracy to capture the full complexity of the syndrome [6,7]. In routine clinical practice, the most useful biomarkers are often those that are rapidly obtainable, repeatable, and already incorporated into standard intensive care work-up, particularly C-reactive protein (CRP) and procalcitonin (PCT), together with complete blood count-derived indices such as leukocyte count and the neutrophil-to-lymphocyte ratio (NLR) [6,8,9]. Their value lies not only in biological relevance but also in practicality, as they can be measured quickly and followed serially during the early phase of illness [8,9]. However, although numerous studies have examined the diagnostic or prognostic role of single biomarker measurements, evidence on whether early temporal trajectories differ between patients with sepsis and those with septic shock remains considerably more limited. The studies identified so far have focused mainly on CRP and PCT and have reported inconsistent results, while comparable longitudinal analyses of simple hematological markers and leukocyte-derived indices remain much less common [10,11,12]. This underexplored area provides the rationale for assessing early changes in routinely available inflammatory markers as a potential aid in distinguishing sepsis from septic shock.

Patient characteristics are also important in determining whether sepsis will progress to septic shock. Chronic comorbid conditions are common in patients with sepsis, and previous studies have shown that underlying diseases may substantially influence both susceptibility to sepsis and subsequent clinical outcomes [13,14]. In parallel, the source of infection appears to be closely related to disease severity, as respiratory and intra-abdominal infections have associated with a higher likelihood more severe stages of sepsis [14,15]. More recent ICU-based data further suggest that septic shock may occur more frequently in patients with abdominal and urinary tract infections, while age, neoplasia, and chronic organ dysfunction contribute to worse prognosis [16]. Nevertheless, most of the available literature has focused either on selected common comorbidities or on the prognostic role of infection foci, whereas studies jointly examining a broader spectrum of comorbidities, source of infection, and routinely available inflammatory biomarkers in relation to the occurrence of septic shock remain limited [14,15,16]. This represents an important gap, as integrating premorbid patient characteristics with practical laboratory markers may improve early risk stratification in patients with sepsis.

Therefore, the present study aimed to investigate which patient-related factors are associated with a higher occurrence of septic shock among patients diagnosed with sepsis, with particular emphasis on comorbidities. Although comorbid conditions are clinically important, their role has not been sufficiently explored in previous research, especially when considered together with other routinely available clinical characteristics. Within this framework, the examined biomarkers were not considered as stand-alone diagnostic tools for establishing sepsis, but rather as routinely available indicators potentially relevant for early risk stratification, severity differentiation, and longitudinal monitoring within an already defined sepsis population. In addition, the study aimed to examine changes in selected biomarkers during the first 72 h after the diagnosis of sepsis. By integrating baseline patient-related clinical factors with early longitudinal changes in routinely available biomarkers, the present study aimed to provide a more practice-oriented framework for early septic shock risk stratification within a sepsis population. Particular focus was placed on markers that are practical, readily available, and commonly used in everyday clinical practice, in order to assess their potential value in the early identification of patients at increased risk of septic shock.

## 2. Methods

### 2.1. Research Design

This study was designed as a retrospective longitudinal observational study involving patients treated in the intensive care unit who were diagnosed with sepsis or septic shock. The study was based on repeated clinical and laboratory follow-up of patients from the time when the diagnosis of sepsis was established according to the SEPSIS-3 criteria. Sepsis was defined as an acute increase in the total SOFA score of 2 points or more, in accordance with the SEPSIS-3 criteria. Accordingly, patient classification in the present study relied on the full SOFA score rather than on the quick Sequential Organ Failure Assessment (qSOFA). Septic shock was defined as sepsis requiring vasopressor therapy to maintain a mean arterial pressure above 65 mmHg, together with a serum lactate level above 2 mmol/L. Patients were included in the study on the day when these criteria were fulfilled, which was also considered the first measurement point.

A broad set of variables was collected for each patient in order to provide a comprehensive clinical profile and to support subsequent analyses of factors associated with septic shock. These variables included demographic and clinical history data, such as comorbidities, surgery status, and the source of infection. In addition, repeated laboratory and clinical measurements were used to monitor early changes in inflammatory and biochemical parameters, including Procalcitonin (PCT), C-reactive protein (CRP), Leukocyte count (LE), percent of Neutrophils, percent of Lymphocytes, Neutrophil-to-lymphocyte ratio (NLR), as well as disease progression during the initial phase after sepsis diagnosis [9,10,16,17,18,19,20].

Particular attention was paid to the first 72 h after diagnosis, during which most laboratory markers were measured at 24 h intervals. The PCT sample was taken on admission, 48 and 72 h after the first sampling. This approach enabled the evaluation of both baseline differences and early longitudinal changes between patients with sepsis and those with septic shock.

The study was approved by the Local Ethics Committee at Valjevo General Hospital, approval OBV-01-9963 dated 14 November 2018. All procedures were conducted in accordance with the ethical standards of the responsible institutional committee and the Declaration of Helsinki.

### 2.2. Data Collection

The observation period covered 12 months, from December 2018 to December 2019 at Valjevo General Hospital, Serbia. A total of 110 patients were initially enrolled. However, the final analytical sample included 106 patients. Four patients were excluded from the final analysis because complete data were unavailable due to errors during sample collection and/or processing. No imputation procedure was applied.

As shown in Table 1, the sample was slightly male-dominated (56.6%), while the largest age subgroup comprised patients aged 75 years or older (41.5%). Most patients underwent surgery (61.3%). Gastrointestinal infection was the most frequently recorded source, whereas respiratory infection was the least represented. Regarding comorbidities, cardiovascular diseases were the most common (69.5%), while the prevalence of most other comorbid conditions was considerably lower. To ensure sufficient representation in subsequent analyses, comorbidities with a prevalence below 15% were excluded from further modeling.

### 2.3. Analysis

The analysis was conducted in three steps. First, baseline demographic, clinical, and laboratory characteristics were compared between patients with sepsis and those with septic shock using univariate tests. Second, variables identified as relevant candidates were entered into a LASSO-assisted logistic regression framework to determine the most informative independent predictors of septic shock. Third, longitudinal changes in inflammatory markers were analyzed using linear mixed-effects models to examine whether their trajectories differed over time according to septic shock status.

Categorical baseline characteristics were compared between patients with sepsis and those with septic shock using the *χ*^2^ test of independence. Continuous predictors (inflammatory and hematological markers) were compared between the two groups using the Mann–Whitney U test. Results were presented descriptively by group, and statistical significance was assessed using *p*-values, with special attention to findings below the conventional threshold of 0.05; values between 0.05 and 0.10 were interpreted as borderline significant.

Because the number of candidate predictors was relatively large in relation to the sample size, the least absolute shrinkage and selection operator (LASSO) approach was used as a variable-selection step before fitting the final logistic regression model. This approach was chosen to reduce model complexity and limit the risk of overfitting in the context of a relatively modest sample size. Specifically, LASSO logistic regression was applied with septic shock as the binary dependent variable, and all candidate clinical predictors were entered simultaneously. This approach shrinks less informative coefficients and retains only variables with the strongest contribution to the model. The optimal penalty parameter was selected using 10-fold cross-validation. After the variable-selection step, a standard binary logistic regression model was fitted, including only the predictors retained by the LASSO approach. Regression coefficients (*β*), standard errors (SE), *p*-values, and odds ratios (*OR*) were estimated for all included variables. Model performance was additionally evaluated in terms of discrimination and calibration. Discrimination was assessed using the area under the receiver operating characteristic curve (AUC), calibration was examined using the Hosmer–Lemeshow goodness-of-fit test, and explained variance was assessed using Cox and Snell R^2^ and Nagelkerke R^2^.

Finally, longitudinal changes in inflammatory markers were analyzed using linear mixed-effects models. Separate models were estimated for each biomarker. Septic shock status, measurement time, and their interaction were entered as fixed effects. To account for repeated observations within the same individual, patient ID was defined as the subject variable, and an unstructured covariance structure was specified for the repeated measurements. The interaction term between septic shock status and measurement time was considered the key indicator of whether marker trajectories differed between groups. A significant interaction was interpreted as evidence of different temporal trends between patients with and without septic shock.

## 3. Results

### 3.1. Nonparametric Tests

Table 2 presents the comparison of baseline demographic and clinical characteristics between patients with sepsis and those with septic shock. Comparisons were performed using the *χ*^2^ test of independence. A statistically significant difference was observed only for gastrointestinal comorbidity, which was significantly less common in the septic shock group. In addition, surgery status showed a borderline significant difference (*p* < 0.10), with a higher proportion of surgical patients in the septic shock group. Although some variation was also observed across Age categories and urologic comorbidity, these differences did not reach statistical significance. No significant between-group differences were found for gender, infection source, and the remaining comorbidities (*p* > 0.10).

Baseline inflammatory markers were compared between patients with sepsis and those with septic shock using the Mann–Whitney U test (Table 3). A statistically significant difference was observed only for procalcitonin (PCT), which was higher in patients with septic shock than in those with sepsis. C-reactive protein (CRP) also showed higher values in the septic shock group, although this difference did not reach statistical significance. No statistically significant between-group differences were found for leukocyte count, neutrophil percentage, lymphocyte percentage, or the neutrophil-to-lymphocyte ratio (NLR).

### 3.2. LASSO Logistic Regression Model

The LASSO procedure reduced the initial set of candidate predictors to four variables with non-zero coefficients. These retained predictors were Age, Surgery, Procalcitonin, and Gastrointestinal comorbidity, and were subsequently entered into the final logistic regression model. Other variables, such as infection source predictors, were evaluated as candidate predictors but were not retained by the LASSO procedure, which is consistent with the absence of significant between-group differences observed in the univariate analysis.

The model was statistically significant overall (*χ*^2^ = 26.50, *p* < 0.001), with Cox and Snell R^2^ and Nagelkerke R^2^ values of 0.221 and 0.300, respectively. These results indicate that the selected predictors jointly contributed to identifying patients with septic shock. The model showed strong discriminative ability, with an AUC of 0.795, indicating excellent performance in distinguishing patients with from those without septic shock. Model calibration was also satisfactory, as indicated by a non-significant Hosmer–Lemeshow goodness-of-fit test (*χ*^2^ = 10.73, *p* = 0.218).

In the final model (Table 4), higher Procalcitonin levels were independently associated with greater odds of septic shock (*β* = 0.021, *OR* = 1.022, *p* = 0.004). By contrast, Gastrointestinal comorbidity was independently associated with lower odds of the outcome (*β* = −2.005, *OR* = 0.135, *p* = 0.011). Although the overall contribution of Age was borderline statistically significant (*p* = 0.069), patients older than 75 years had significantly higher odds of septic shock compared to the reference category (*β* = 1.229, *OR* = 3.417, *p* = 0.025). In contrast, Surgery was retained by the LASSO procedure but was not independently associated with septic shock in the final model (*β* = 0.550, *OR* = 1.733, *p* = 0.274).

### 3.3. Longitudinal Trends of Inflammatory Markers According to Septic Shock Status

**Figure 1:**
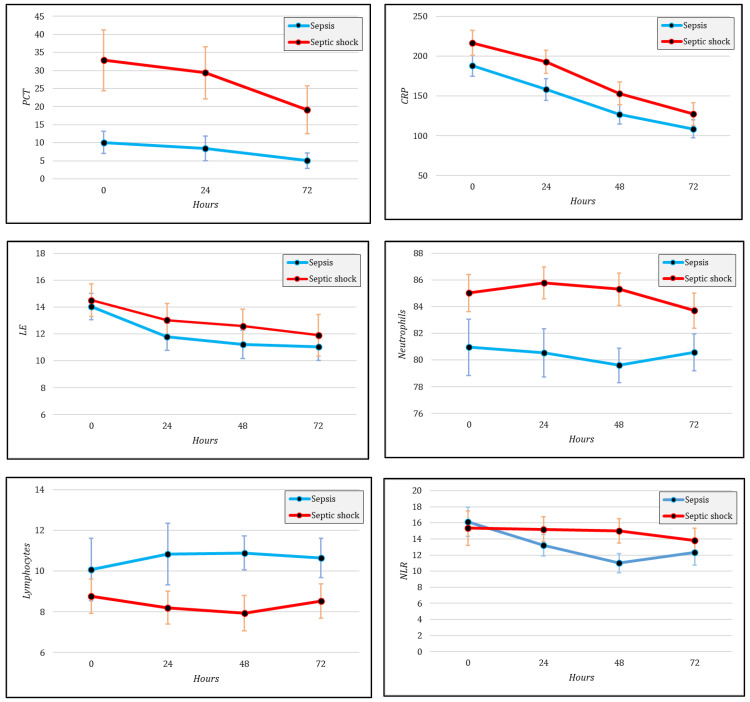
Longitudinal trajectories of inflammatory markers across repeated measurements.

The longitudinal changes in inflammatory markers according to septic shock status are illustrated in Figure 1. These figures present the estimated mean values of each parameter across repeated measurements for patients with sepsis and those with septic shock, allowing a visual comparison of temporal patterns between the two groups.

Table 5 summarizes the group × time interaction terms derived from linear mixed-effects models for repeated inflammatory markers, stratified by septic shock status. A statistically significant interaction was identified only for neutrophil percentage (*F* = 2.801, *p* = 0.044), suggesting that changes over time in this parameter differed between patients with sepsis and those with septic shock. Specifically, the estimated trajectories shown in Figure 1 indicate an early increase in neutrophil percentage during the first 24 h in the septic shock group, whereas in the sepsis group the values decreased during the first 48 h. This divergence suggests that neutrophil dynamics may provide more clinically relevant longitudinal information than single baseline hematological measurements in the early phase after diagnosis. No statistically significant interaction effects were observed for procalcitonin, C-reactive protein, leukocyte count, lymphocyte percentage, or the neutrophil-to-lymphocyte ratio, indicating comparable temporal patterns across the two groups for these markers.

## 4. Discussion

### 4.1. Principal Findings

The present study yielded three principal findings. First, among the baseline inflammatory markers, only PCT was significantly higher in patients with septic shock than in those with sepsis, whereas CRP showed only a borderline difference. This is broadly consistent with previous studies showing that PCT has better discriminatory value than CRP for identifying more severe forms of sepsis [10,21]. However, this finding should be interpreted with caution, since PCT is a supportive rather than stand-alone biomarker, its specificity is limited, and serum levels may also increase in non-infectious inflammatory conditions [22,23]. This point is particularly relevant in predominantly surgical ICU populations such as ours, because postoperative inflammatory responses may also influence PCT levels [24].

Second, in the logistic regression model, older age, particularly age above 75 years, were associated with greater odds of septic shock. The association with older age is in line with previous evidence indicating that elderly patients are more vulnerable to severe sepsis because of reduced physiological reserve, immunosenescence, and a greater burden of chronic disease [25,26]. In contrast, surgery did not remain independently associated with septic shock after adjustment, suggesting that its crude association may have reflected the influence of other correlated clinical factors.

Third, during the first 72 h, only neutrophil percentage showed a significantly different temporal pattern between the two groups, with an early increase in septic shock and an early decrease in sepsis. This finding supports the view that dynamic neutrophil behavior may reflect differences in the early host response even when single baseline hematological measurements appear similar. From a clinical perspective, this pattern may suggest that repeated assessment of simple differential blood count parameters could offer additional short-term stratification value beyond isolated admission values. At the same time, the absence of significant longitudinal differences for most other markers is not entirely unexpected, because previous studies that examined serial biomarker changes in sepsis and septic shock have also mainly emphasized PCT and CRP, with less consistent evidence for simple blood-count-derived indices [10,11,27]. Overall, our findings suggest that early recognition of septic shock may benefit from the combined interpretation of selected routine biomarkers and patient-related characteristics, although the present biomarker signals were limited primarily to baseline PCT differences and the early trajectory of neutrophil percentage.

### 4.2. Comorbidities and the Occurrence of Septic Shock

One of the most unexpected findings of the present study was the inverse association between gastrointestinal comorbidity and the occurrence of septic shock. Namely, gastrointestinal comorbidity was significantly less frequent among patients with septic shock. The available literature suggests that this finding may reflect differences in host response rather than differences in infectious burden alone. The gut has long been regarded as an important driver of critical illness, while growing evidence indicates that gut barrier dysfunction and microbiota alterations can shape systemic immunity and influence the course of sepsis [28,29,30]. In this context, chronic gastrointestinal disorders may be associated with repeated low-grade exposure to microbial products, potentially promoting endotoxin tolerance or broader forms of innate immune reprogramming that attenuate an excessive hyperinflammatory response once sepsis occurs [31,32]. This interpretation is also compatible with the concept of tissue tolerance, according to which survival during severe infection depends not only on pathogen clearance but also on the host’s ability to limit inflammation-induced organ injury [33]. However, these mechanisms remain speculative in the context of the present data and should not be interpreted as direct explanations of the observed association. Alternative explanations should also be considered, including residual confounding, bias related to the retrospective study design, and the broad heterogeneity of the gastrointestinal comorbidity category. At the same time, no direct markers of gut barrier dysfunction, microbial translocation, or immune reprogramming were available in this study. Accordingly, this finding should be viewed as hypothesis-generating rather than definitive, while still supporting further investigation of the gut–immune axis as a possible modifier of septic shock risk.

A more cautious interpretation is required for insulin-dependent diabetes. Patients with insulin-dependent diabetes appeared more frequently in the septic shock group, but this difference did not reach statistical significance. Therefore, this finding should be considered only indicative. The relationship between diabetes and sepsis outcomes remains inconsistent in the literature: an updated meta-analysis found that diabetes was not clearly associated with worse overall survival in sepsis, although it was linked to a higher risk of acute renal failure, while high glucose levels at admission were associated with worse in-hospital outcomes [34]. Reviews on diabetes and sepsis also suggest that chronic hyperglycemia, immune dysfunction, endothelial injury, and metabolic dysregulation may modify the host response to infection, even though the net clinical effect is not uniform across studies [35]. In that sense, insulin dependence in our study may reflect more advanced metabolic burden or poorer physiologic reserve. However, because of the small number of such patients in our sample, no firm conclusion can be drawn.

A similarly careful interpretation applies to urologic comorbidity. Although urologic comorbidity was numerically more common among patients with septic shock, the association was not statistically significant and should therefore be regarded as a hypothesis-generating signal only. This pattern is nevertheless compatible with literature showing that urinary tract pathology can be linked with more severe septic presentations, especially when accompanied by obstruction, recent instrumentation, bacteremia, or functional urinary abnormalities [36,37,38]. At the same time, our variable captured broad pre-existing urologic comorbidity rather than acute urosepsis-specific risk factors such as obstruction or bacteremia. For that reason, it is more appropriate to interpret this finding as a possible marker of underlying vulnerability than as evidence of an independent causal relationship.

Taken together, these findings suggest that the comorbidity profile may contribute to the heterogeneity of septic patients, but not all signals should be interpreted with the same level of certainty. In our study, the inverse association for gastrointestinal comorbidity was the most robust and statistically supported finding, whereas the patterns observed for insulin-dependent diabetes and urologic comorbidity should be viewed as preliminary indications that warrant evaluation in larger and more clinically stratified cohorts.

### 4.3. Clinical Interpretation and Implications

The findings of the present study have several potential clinical implications. First, they support the value of routinely available biomarkers in the early assessment of patients with sepsis. In everyday intensive care practice, markers such as procalcitonin, CRP, leukocyte count, and differential blood count are attractive because they are inexpensive, rapidly obtainable, and can be repeatedly measured without additional diagnostic burden. However, these markers should be interpreted cautiously and always in conjunction with the overall clinical picture [22,23]. In the present study, their clinical relevance should be understood primarily in terms of early risk stratification and temporal monitoring, rather than as stand-alone diagnostic tools or independent guides for therapeutic decision-making. In particular, although PCT was the only biomarker independently associated with septic shock in our cohort, it should not be regarded as a stand-alone indicator, since its specificity is limited and serum levels may also increase in non-infectious inflammatory conditions, including trauma and postoperative inflammatory states [39]. In addition, PCT should not be interpreted as a decisive tool for antimicrobial decision-making on its own, but rather as an adjunct to clinical assessment in selected contexts. This caution is especially relevant in our cohort, in which most patients had undergone surgery. Overall, the present findings support the contribution of routine biomarkers to early risk stratification, but not the use of PCT alone to guide diagnosis or therapy.

Second, the present findings indicate that patient-related factors should not be overlooked in the early stratification of septic patients. Age and comorbidity profile appeared to contribute to the heterogeneity of septic presentations, suggesting that the risk of septic shock cannot be understood solely through biomarker values. In this context, the inverse association observed for gastrointestinal comorbidity may be clinically relevant, not because it immediately changes practice, but because it points to the possibility that pre-existing host factors influence progression from sepsis to septic shock. However, alternative explanations, including residual confounding and the heterogeneous nature of the gastrointestinal comorbidity category, should also be taken into account. At the same time, the patterns observed for insulin-dependent diabetes and urologic comorbidity should be interpreted only as preliminary signals. Also, infection source did not show a clear independent signal in the present cohort, as no significant between-group differences were observed in the initial comparisons and the corresponding variables were not retained in the final multivariable model.

An important contribution of the present study lies in combining baseline clinical characteristics with early biomarker trajectories, rather than relying on either static laboratory values or patient-related factors alone. Taken together, our results suggest that early recognition of septic shock may benefit from an integrated approach combining routine laboratory markers with readily available clinical information, rather than relying on isolated biomarker measurements alone. Such an approach may be particularly useful in resource-constrained clinical settings, where rapid and widely accessible indicators are needed to support bedside decision-making. In addition, future research may benefit from evaluating whether newer readily available hematological parameters, such as monocyte distribution width (MDW), provide incremental value beyond conventional routine biomarkers [40,41,42,43].

## 5. Conclusions

In conclusion, this study suggests that the early recognition of septic shock among patients with sepsis may benefit from the combined assessment of routinely available biomarkers and patient-related clinical characteristics. Among the examined laboratory parameters, procalcitonin showed the strongest association with septic shock, while neutrophil percentage demonstrated a distinct early temporal pattern. In addition, gastrointestinal comorbidity emerged as an unexpected factor inversely associated with septic shock, whereas insulin-dependent diabetes and urologic comorbidity showed only indicative signals. These findings support the clinical value of practical and accessible indicators in early risk stratification, while also highlighting the need for further validation in larger and more diverse cohorts.

Several limitations should be acknowledged. This was a single-center retrospective study with a relatively limited sample size, which restricts the generalizability of the findings and reduces the precision of subgroup analyses. Although LASSO was used to reduce the number of candidate predictors and limit model complexity, the relatively modest sample size may still have affected model stability and increased the risk of overfitting. The examined comorbidity groups were also heterogeneous, particularly the gastrointestinal category, which limits pathophysiological interpretation. In addition, not all biomarkers were measured with the same temporal resolution, since procalcitonin was not assessed 24 h after ICU admission because of resource limitations. The study focused on routine laboratory and clinical parameters and did not include mechanistic markers related to immune regulation, gut barrier dysfunction, or microbial translocation, which might have helped explain some of the observed associations. In addition, because the full SOFA score and serum lactate were used as part of the SEPSIS-3-based classification framework, the present study did not examine their additional predictive value beyond case definition. Also, this study did not include emerging biomarkers such as presepsin, MR-proADM, or sTREM-1, nor newer hematological parameters such as monocyte distribution width (MDW), as these markers were not available in the retrospective dataset. Although such markers may provide additional diagnostic or prognostic information, the present study was intentionally focused on routinely available laboratory indicators that were consistently collected in everyday ICU practice. In addition, the cohort included a high proportion of surgical patients, which may limit the generalizability of the biomarker findings and is particularly relevant when interpreting procalcitonin levels in the early phase after diagnosis. Finally, although the cohort was collected approximately seven years ago, the data still provide a relevant representation of patient status in that period and remain informative for evaluating the early application of the Sepsis-3 protocol.

Future studies should therefore aim to validate these findings in larger, multicenter cohorts and to examine whether combining routine biomarkers with a more refined characterization of comorbidities and infection source improves early prediction of septic shock. Particular attention should be given to the potential role of gastrointestinal comorbidity, which emerged as the most distinctive comorbidity-related finding in the present study but requires confirmation in better stratified populations and in studies with more detailed biological profiling. Future studies should therefore examine whether combining routine biomarkers with emerging biomarkers and newer CBC-derived parameters, such as presepsin, MR-proADM, sTREM-1, or MDW, improves early risk stratification in patients with sepsis and septic shock. Given that this study was conducted on a relatively small sample collected at a single center, future research should consider incorporating knowledge from previous studies through advanced analytical tools (e.g., a Bayesian approach).

## Figures and Tables

**Table 1:** Sample profile.

Variables	Frequency	Percentage
Gender		
Males	60	56.6%
Females	46	43.4%
Age		
<65	38	35.8%
65–74	24	22.6%
≥75	44	41.5%
Septic status		
Sepsis	65	61.3%
Septic shock	41	38.7%
Surgery		
No	41	38.7%
Yes	65	61.3%
Infection source		
Gastrointestinal	50	47.2%
Trauma	19	17.9%
Respiratory	9	8.5%
Other	38	35.8%
Comorbidity		
Cardiovascular	73	69.5%
Vascular	6	5.7%
Respiratory	24	22.6%
Malignancy	21	19.8%
Non-insulin-dependent diabetes	19	17.9%
Insulin-dependent diabetes	10	9.4%
Endocrine	11	10.4%
Neurologic	11	10.4%
Rheumatologic	2	1.9%
Psychiatric	11	10.4%
Gastrointestinal	23	21.7%
Urologic	22	20.8%

**Table 2:** Comparison of demographic and clinical characteristics between patients with sepsis and septic shock.

Variables	Sepsis	Septic Shock	Significance
Gender			
Males	39	21	*p* = 0.492
Females	26	20	
Age			
<65	28	10	*p* = 0.131
65–74	14	10	
≥75	23	21	
Surgery			
No	30	11	*p* = 0.074
Yes	35	30	
Infection source = Gastrointestinal			
No	34	16	*p* = 0.257
Yes	31	25	
Infection source = Respiratory			
No	59	38	*p* = 0.731
Yes	6	3	
Infection source = Trauma			
No	55	32	*p* = 0.550
Yes	10	9	
Comorbidity = Cardiovascular			
No	22	10	*p* = 0.386
Yes	42	31	
Comorbidity = Respiratory			
No	50	32	*p* = 0.893
Yes	15	9	
Comorbidity = Non-insulin-dependent diabetes			
No	53	32	*p* = 0.850
Yes	12	9	
Comorbidity = Insulin-dependent diabetes			
No	56	31	*p* = 0.263
Yes	9	10	
Comorbidity = Gastrointestinal			
No	45	38	*p* = 0.009
Yes	20	3	
Comorbidity = Urologic			
No	55	29	*p* = 0.141
Yes	10	12	

**Table 3:** Comparison of baseline inflammatory markers between patients with sepsis and septic shock.

Inflammatory Markers	Sepsis	Septic Shock	Significance
Procalcitonin—PCT	10.20	32.82	*p* = 0.001
C-reactive protein—CRP	187.13	216.68	*p* = 0.094
Leukocyte count—LE	13.68	14.52	*p* = 0.724
Neutrophil (%)	80.96	85.02	*p* = 0.174
Lymphocyte (%)	10.07	8.77	*p* = 0.919
Neutrophil-to-lymphocyte ratio—NLR	16.14	15.33	*p* = 0.893

**Table 4:** Logistic regression model.

Variable	*β*	SE	*p*	*OR*	*OR* (95% CI)	
Age (base: <65)						
65–74	1.021	0.626	0.103	2.777	0.814	9.469
75≤	1.229	0.547	0.025	3.417	1.168	9.990
Surgery	0.550	0.503	0.274	1.733	0.647	4.647
Procalcitonin	0.021	0.007	0.004	1.022	1.007	1.037
Gastrointestinal comorbidity	−2.005	0.791	0.011	0.135	0.061	0.579
Constant	−1.673	0.575	0.004			

**Table 5:** Summary of septic shock status × time interaction effects from linear mixed-effects models for repeated inflammatory markers.

Inflammatory Markers	F-Statistic	*p*
Procalcitonin—PCT	0.214	0.807
C-reactive protein—CRP	1.352	0.262
Leukocyte count—LE	1.918	0.131
Neutrophil (%)	2.801	0.044
Lymphocyte (%)	1.263	0.293
Neutrophil-to-lymphocyte ratio—NLR	0.868	0.461

## Data Availability

Data is unavailable due to privacy or ethical restrictions.

## Abbreviations

The following abbreviations are used in this manuscript:

CRP	C-reactive protein
LE	Leukocyte count
NLR	Neutrophil-to-lymphocyte ratio
PCT	Procalcitonin
